# Impact of COVID‐19 versus chronic rhinosinusitis/rhinitis associated olfactory dysfunction on health utility and quality of life

**DOI:** 10.1002/lio2.921

**Published:** 2022-09-24

**Authors:** Thanh Luong, Sophie S. Jang, Mena Said, Adam S. DeConde, Carol H. Yan

**Affiliations:** ^1^ Department of Otolaryngology – Head and Neck Surgery University of California San Diego San Diego California USA

**Keywords:** chronic rhinosinusitis, COVID‐19, health utility values, olfactory dysfunction, quality of life

## Abstract

**Background:**

Olfactory dysfunction (OD) is associated with both post‐viral and inflammatory etiologies such as COVID‐19 and chronic rhinosinusitis/rhinitis (CRS/R) respectively, to result in reduced quality of life (QoL). However, the former typically induces a sudden‐onset OD while the latter has a gradual presentation. This study aims to establish and compare health utility values (HUVs) and olfactory‐specific QoL measurements between patients with COVID‐19 and CRS/R related OD.

**Methods:**

This prospective study surveyed COVID‐19 and CRS/R patients with self‐reported OD using HUV assessments (EuroQol‐visual analog scale [EQ‐VAS], EuroQol‐5 dimension [EQ‐5D], time trade‐off [TTO]) and olfactory and sinonasal QoL measures (questionnaire of olfactory disorders –negative and positive statements [QOD‐NS + PS] and sino‐nasal outcome test [SNOT‐22]). A subgroup of subjects completed objective olfactory testing. Intergroup mean scores were compared using Mann–Whitney U tests.

**Results:**

One hundred eleven subjects were enrolled: mean age ± SD (43.0 ± 15.4 years), 55.9% female. CRS/R was associated with lower HUVs as measured by EQ‐VAS (CRS/R: 0.67 ± 0.18 vs. COVID‐19: 0.74 ± 0.19, *p* = .03) and worse SNOT‐22 scores in both overall (CRS/R: 49.03 ± 21.04 vs. COVID‐19: 27.58 ± 18.45, *p* < .001) and subgroup analysis of objectively confirmed OD subjects (CRS/R: 52.40 ± 22.78 vs. COVID‐19: 29.84 ± 21.10, *p* = .01). On the other hand, COVID‐19 has greater burden on olfactory‐specific QoL (QOD‐NS + PS, COVID‐19: 23.19 ± 13.73 vs. CRS/R: 17.25 ± 11.38, *p* = .04). Both groups demonstrated a similar decrease in health using the EQ‐5D assessment.

**Conclusion:**

CRS/R associated OD has a more severe impact on general health and sinonasal specific QoL outcomes, while COVID‐19 associated OD has a greater burden on olfactory‐specific QoL.

**Level of evidence:**

Level 2c.

## Graphical abstract

This work compared multiple quality of life instruments associated with olfactory dysfunction in patients with chronic rhinosinusitis/rhinitis and COVID‐19. Utilizing health utility and olfactory‐specific quality of life measurements, our findings showed that chronic rhinosinusitis/rhinitis induced olfactory dysfunction had a more severe impact on general health utility and sinonasal specific quality of life outcomes, while COVID‐19 induced olfactory dysfunction had a greater burden on olfactory‐specific quality of life.

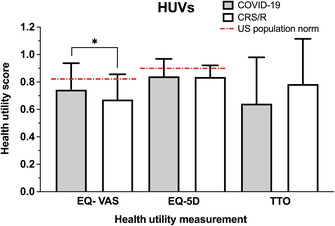

## 1 INTRODUCTION

Olfactory dysfunction (OD) affects one in five adults and is associated with increased mortality and decreased quality of life (QoL).^1^ Through the COVID‐19 pandemic, post‐viral OD associated with COVID‐19 infection has become well recognized, manifesting as a profound and acute onset chemosensory loss.^2^, ^3^ The rate of persistent post‐COVID OD, when defined as over 60 days of symptoms, is roughly 19%^4^ and accounts for the increasing number of patients who are seeking medical help for OD. Another common cause of olfactory impairment seen in the otolaryngology clinics is inflammatory drive due to chronic rhinosinusitis (CRS) and rhinitis (R). Although olfactory sensitivity and distorted odor perception in patients with rhinitis are often less severe than in patients with CRS, both groups (CRS/R) present with gradual onset, fluctuating olfactory symptoms, in contrast to the acute presentation of OD associated with COVID‐19 infection.

Health utility values (HUVs) are self‐assessed health related general QoL measurements often used by health economists to measure a person's valuation of being in a particular health state.^5^ HUVs also allow for comparison of the QoL impact across different disease states, which is particularly useful in cost–utility analyses of pharmaceutical and other healthcare interventions. Health values in CRS and in persistent OD associated with COVID‐19 have been characterized in previous studies.^6^, ^7^, ^8^ However, the HUVs associated specifically with patients with CRS/R related OD have not been described. This study aims to (1) establish HUVs in CRS/R related OD and (2) compare HUVs and olfactory and sinonasal QoL measures between patients with COVID‐19 and CRS/R related OD.

## 2 MATERIALS AND METHODS

### 2.1 Subject recruitment

Institutional Review Board approval by UC San Diego (UCSD) was obtained for this prospective study (IRB 191951 and IRB 200485X). This study recruited PCR‐confirmed COVID‐19 patients from UCSD and patients who presented to UCSD Rhinology clinics between June 2021 and February 2022. Inclusion criteria includes English speaking adults (age > 18 years old) with PCR‐confirmed history of COVID‐19 with OD or with a history of CRS/R with OD. Subjects with both etiologies of OD were excluded. Informed consent was obtained from all subjects. All subjects completed an electronic health survey via Qualtrics (Provo, Utah) which included baseline information about their smell function, clinical history, demographics, and health utility assessments olfactory and sinonasal QoL measures. A subgroup of subjects completed objective olfactory testing using either the University of Pennsylvania smell identification test or the brief smell identification test (UPSIT/BSIT).

### 2.2 Health utility value assessments

#### 2.2.1 EuroQol‐visual analog scale

Participants subjectively rated their own health status using a sliding scale ranging from 0 (worst imaginable health) to 100 (best health). Each EuroQol‐visual analog scale (EQ‐VAS)‐based health utility score was determined by dividing the selected value by 100.^9^

#### 2.2.2 EuroQol‐5 dimension

The EuroQol‐5 dimension (EQ‐5D) measures general QoL and consists of five domains: motility, self‐care, usual activities, pain/discomfort, and anxiety/depression. Subjects' answer to each domain is assigned a value based on severity: no problem (1), slight problems (2), moderate problems (3), severe problems (4), or extreme problems (5). The “EQ‐5D‐5L Crosswalk Index Value Calculator” was used to convert each participant's responses into a single value ranges from 0 (death) to 1 (best health possible).^9^

#### 2.2.3 Time trade‐off

Participants were asked “Imagine you have 20 years left to live with complete smell loss or you can give up some years to live and have complete normal smell. In your mind, living how many years with normal smell is equivalent to living 20 years with complete smell loss?” Time trade‐off (TTO) score was the ratio of the number of years indicated over 20.^10^

### 2.3 Olfactory‐specific quality of life assessments

#### 2.3.1 Visual analog scale

Participants subjectively rated their current sense of smell using a sliding scale from 0 (completely gone) to 10 (completely normal). Visual analog scale (VAS) scores were converted to a 100‐point scale by multiplying the selected values by 10. Score ranges from 0 (no smell) to 100 (normal smell).^11^

#### 2.3.2 Questionnaire of olfactory disorders‐17 negative and two positive statements [QOD‐NS + PS]

Questionnaire of OD (QOD) is a validated olfactory‐specific QoL instrument used for assessment of subjective severity of OD.^12^ Participants were asked 17 negative statements (NS) to assess the degree of suffering from olfactory impairment, and two positive statements (PS) to determine how well they are coping with the impairment.^12^ Answer choices included four options: agree, partly agree, partly disagree, or disagree, corresponding to a score of 3 to 0 (for negative statements) or 0 to 3 (for positive statement) points, respectively. The sum of the scores for QOD‐NS and QOD‐PS makes the total QOD‐NS + PS score with a range from 0 to 57. Higher scores indicate a stronger impairment.^12^

#### 2.3.3 Sinonasal outcome test

Sinonasal outcome test (SNOT‐22) is a validated CRS‐specific QoL instrument with 22 questions that aim to assess the presence and severity of CRS symptoms.^13^ Total SNOT‐22 scores range from 0 to 110.^13^ The rhinologic symptoms domain includes symptoms of decreased smell/decreased sense of taste, need to blow nose, sneezing, runny nose, thick nasal discharge, and blockage/congestion of the nose.^14^ Rhinologic symptoms domain scores range from 0 to 30. Higher scores indicating worse functioning or symptom severity.^13^

### 2.4 Quantitative measurements of olfactory function

#### 2.4.1 University of Pennsylvania smell identification test

The University of Pennsylvania smell identification test (UPSIT) is a standardized 40‐item instrument used to assess an individual's ability to detect odors and effectively detect olfactory dysfunction.^15^ Scores were classified as: 35–40 (normal), 31–34 (mild hyposmia), 26–30 (moderate hyposmia), 19–25 (severe hyposmia), 6–18 (anosmia), and <6 (malingering).^16^

#### 2.4.2 The brief smell identification test

This test is an abbreviated, 12‐item version of the UPSIT.^17^ Scores were classified as: 10–12 (normal), 6–9 (mild hyposmia), 3–5 (moderate hyposmia), and 0–2 (severe hyposmia).^18^, ^19^

### 2.5 Statistical analysis

Statistical analysis was performed with SPSS (IBM, Armork, NY) and Prism version 9.3.1 (GraphPad, San Diego, CA). Chi‐squared analysis and Mann–Whitney U tests were used to compare distribution across qualitative and quantitative data, respectively. Spearman's correlation analysis was performed to determine the relationship among different QoL measurements. Multivariate linear regression analysis was conducted to adjust for potential confounding variables, which have been reported in literature to affect QoL assessment. All tests were two‐tailed and statistical significance was considered for *p* < .05.

## 3 RESULTS

### 3.1 Demographics

**FIGURE 1:**
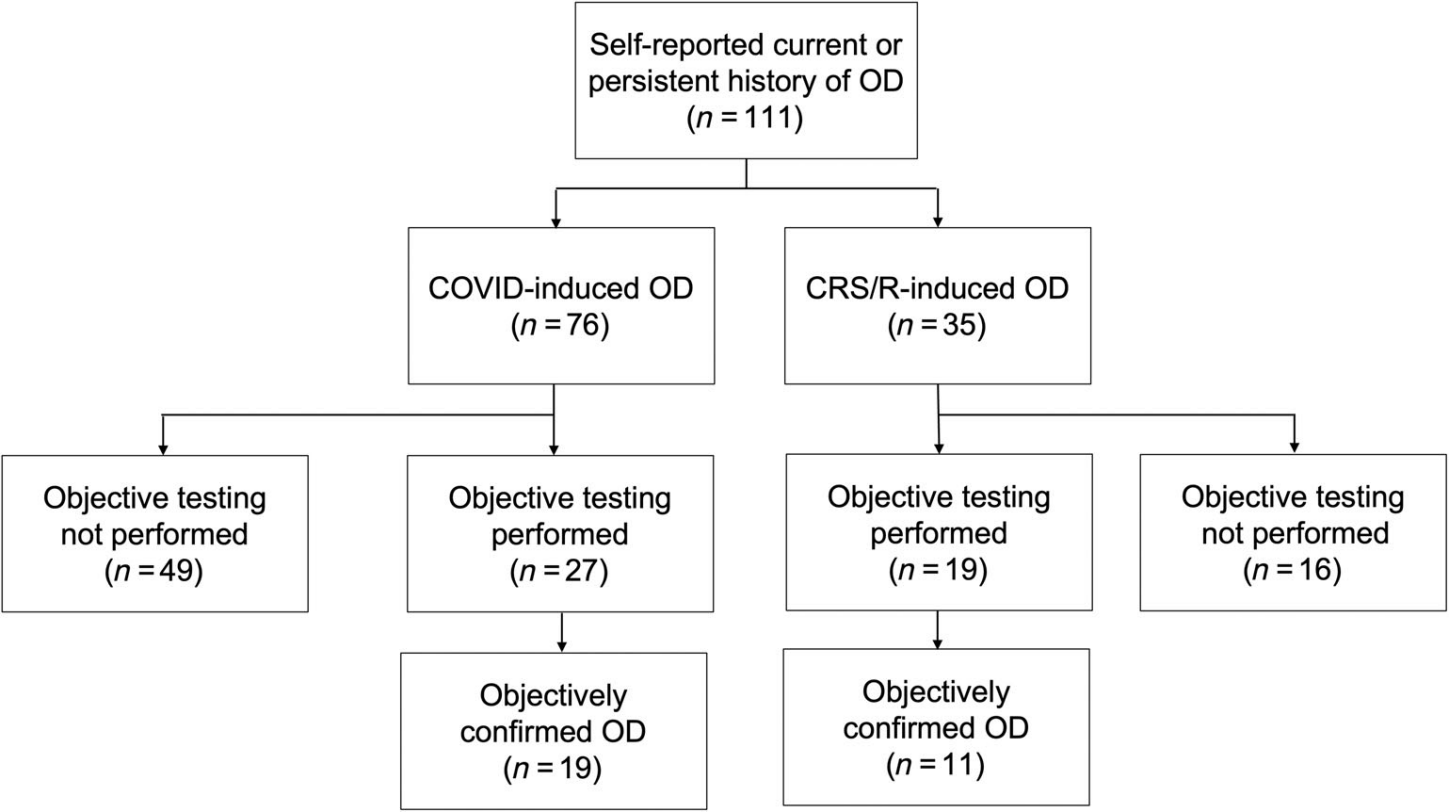
Flow diagram depicting recruitment summary. OD, olfactory dysfunction

This study prospectively recruited 111 subjects with self‐reported OD: 76 (68.5%) due to COVID‐19 and 35 (31.5%) due to CRS/R (Figure 1). Objective smell tests (UPSIT/BSIT) were completed by a subset of the subjects (*n* = 46, 41.4%). Demographic data and clinical characteristics of the participants are summarized in Table 1. Notably, the CRS/R participants were older (47.0 ± 14.0 vs. 41.1 ± 15.8, *p* = .03) and more frequently reported duration of OD to be >12 months (*p* = .04). There was no difference in gender, race, or past medical history between the two groups.

**TABLE 1:** Participants demographic data

	Total (*n* = 111)	Self‐reported OD etiology		*p*‐Value
		COVID‐19 (*n* = 76)	CRS/R (*n* = 35)	
Gender, *n* (%)				
Female	62 (55.9)	44 (57.9)	18 (51.4)	.63
Male	49 (44.1)	32 (42.1)	17 (48.6)	.69
Other	0 (0.0)	0 (0.0)	0 (0.0)	NA
Age, mean in years (SD)	43 (15.4)	41.1 (15.8)	47.0 (14.0)	.03*
Race, *n* (%)				
Hispanic	18 (16.2)	15 (19.7)	3 (8.6)	.20
White, non‐Hispanic	69 (62.3)	47 (61.8)	22 (62.9)	.94
Black, non‐Hispanic	1 (0.9)	1 (1.3)	0 (0.0)	.50
Asian or Pacific Islander	15 (13.5)	8 (10.5)	7 (20.0)	.23
American Indian or Alaskan Native	0 (0.0)	0 (0.0)	0 (0.0)	NA
Two or more races	8 (7.2)	5 (6.6)	3 (8.6)	.71
Duration of smell loss, *n* (%)				
<1 month ago	43 (38.7)	40 (52.6)	3 (8.6)	.15
1–3 months ago	8 (7.2)	7 (9.2)	1 (2.9)	.84
4–6 months ago	11 (9.9)	8 (10.5)	3 (8.6)	.93
6–9 months ago	14 (12.6)	12 (15.8)	2 (5.7)	.72
9–12 months ago	7 (6.3)	4 (5.3)	3 (8.6)	.87
>12 months ago	26 (23.4)	4 (5.3)	22 (62.9)	.04*

*Note*: *p < .05; **p < .01 (two‐tailed).

Abbreviations: CRS/R, chronic rhinosinusitis or rhinitis; NA, non applicable; OD, olfactory dysfunction; PMH, past medical history; SD, standard deviation.

### 3.2 Health utility values

**FIGURE 2:**
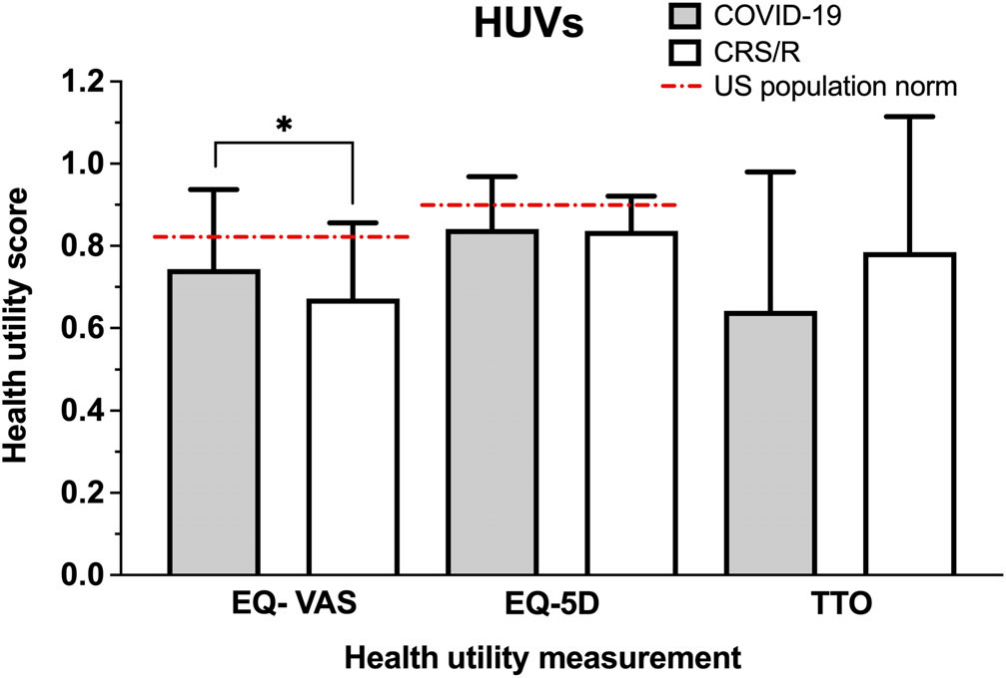
Comparison of mean health utility values between COVID‐19 and CRS/R groups among all subjects with self‐reported OD. Age‐matched US population norms VAS: 0.82, EQ‐5D: 0.85.^19^ *Y*‐axis = health utility score. CRS/R, chronic rhinosinusitis or rhinitis; EQ‐5D, EuroQol‐5 dimension; OD, olfactory dysfunction; VAS, visual analog scale. *p < .05

Using three different measurements of health state, the measured HUVs (mean ± SD) in each group were (1) COVID‐19, EQ‐VAS: 0.74 ± 0.19, EQ‐5D: 0.84 ± 0.13, TTO: 0.64 ± 0.34 and (2) CRS/R, EQ‐VAS: 0.67 ± 0.18, EQ‐5D: 0.84 ± 0.08, TTO: 0.78 ± 0.33 **(**Table 2 **).** Overall, the perceived health status of subjects with COVID‐19 and CRS/R associated OD were lower compared with previously reported age‐matched population norms in the United States (EQ‐VAS: 0.82, EQ‐5D: 0.85)^20^ **(**Figure 2 **)**. When comparing the health status between the COVID‐19 and CRS/R groups using an indirect health measurement such as EQ‐5D, there was no significant difference in mean HUV between the two groups (EQ‐5D, COVID‐19: 0.84 ± 0.13 vs. CRS/R: 0.84 ± 0.08, *p* = .31). However, subjects with CRS/R induced OD reported lower health utility scores using EQ‐VAS, a direct assessment of health status relating to OD, compared to those with COVID‐19 associated OD (CRS/R: 0.67 ± 0.18 vs. COVID‐19: 0.74 ± 0.19, *p* = .03). Conversely, when utilizing other direct health measurements such as TTO, the COVID‐19 cohort had lower health scores compared to the CRS/R cohort that approached statistical significance (COVID‐19: 0.64 ± 0.34 vs. CRS/R: 0.78 ± 0.33, *p* = .06).

**TABLE 2:** Comparisons of overall VAS, HUVs, and olfactory‐specific QoL

	COVID‐19 (*n* = 76)	CRS/R (*n* = 35)	*p*‐Value
VAS, mean (SD)	43.06 (32.79)	34.29 (25.88)	.35
HUV, mean (SD)			
EQ‐VAS	0.74 (0.19)	0.67 (0.18)	.03*
EQ‐5D	0.84 (0.13)	0.84 (0.08)	.31
TTO	0.64 (0.34)	0.78 (0.33)	.06
Olfactory‐specific QoL, mean (SD)			
QOD‐NS + PS	23.18 (13.73)	17.25 (11.38)	.04*
SNOT‐22	27.58 (18.45)	49.03 (21.04)	<.001**
SNOT‐22 rhinologic domain	6.48 (4.26)	15.42 (6.03)	<.001**

*Note*: *p < .05; **p < .01 (two‐tailed).

Abbreviations: CRS/R, chronic rhinosinusitis or rhinitis; EQ‐VAS, EuroQol‐Visual Analog Scale; EQ‐5D, EuroQol‐5 dimension; HUV, health utility value; QOD‐NS + PS, questionnaire of olfactory disorders negative statements and positive statements; SNOT‐22, sino‐nasal outcome test; SD, standard deviation; TTO, time trade‐off; VAS, visual analog scale of subjective olfaction.

**FIGURE 3:**
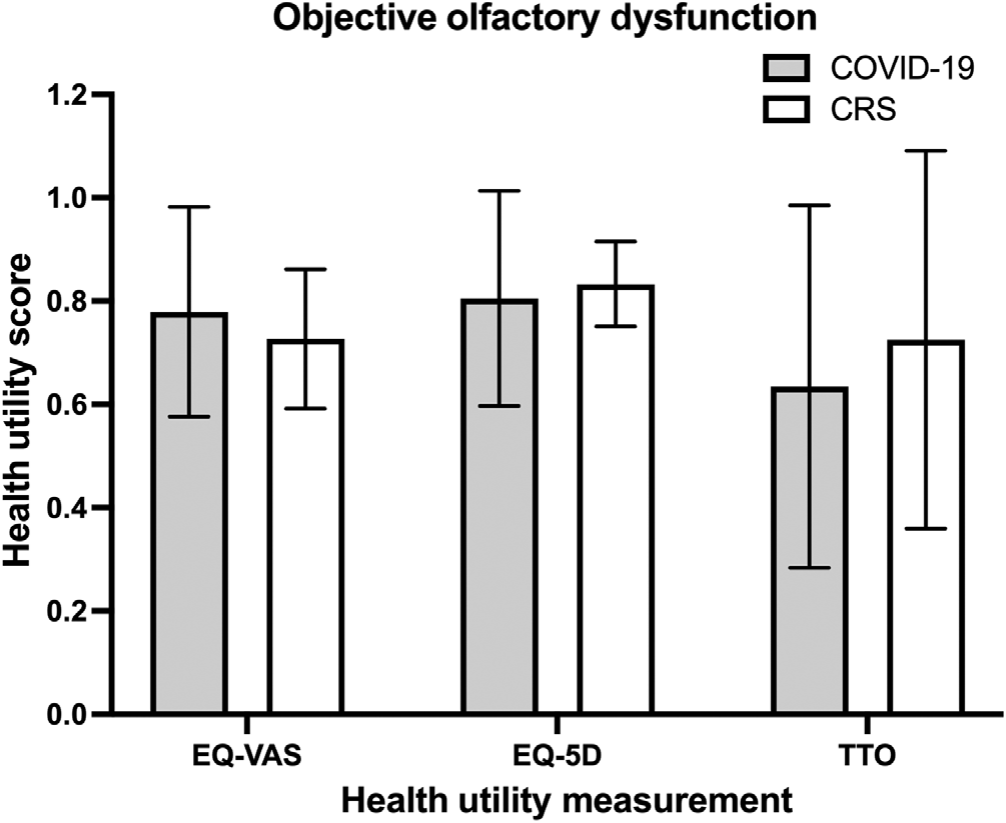
Comparison of mean health utility values between COVID‐19 and CRS/R groups among subjects with quantifiably measured OD as confirmed by UPSIT or BSIT. *Y*‐axis = health utility score. CRS/R, chronic rhinosinusitis or rhinitis; OD, olfactory dysfunction; UPSIT, University of Pennsylvania smell identification test; BSIT, brief smell identification test

Of the 46 participants who underwent objective testing for OD, 30 of them (65.2%) had measurable OD (UPSIT ≤34 or BSIT ≤9) (Figure 1). Subgroup analysis was performed utilizing only the subjects with objectively confirmed OD as depicted in Figure 3. Compared to the entire cohort, those with objectively measurable OD reported similar overall health scores: (1) COVID‐19, EQ‐VAS: 0.78 ± 0.20, EQ‐5D: 0.81 ± 0.21, TTO: 0.63 ± 0.35 and (2) CRS/R, EQ‐VAS: 0.73 ± 0.13, EQ‐5D: 0.83 ± 0.08, TTO: 0.73 ± 0.37 (Table 3).

**TABLE 3:** Sub‐analysis comparisons of VAS, HUVs, and Olfactory‐specific QoL among those with objectively confirmed OD

	COVID‐19 (*n* = 19)	CRS/R (*n* = 11)	*p*‐Value
VAS, mean (SD)	41.67 (28.75)	30.00 (25.39)	.38
HUV, mean (SD)			
EQ‐VAS	0.78 (0.20)	0.73 (0.13)	.11
EQ‐5D	0.81 (0.21)	0.83 (0.08)	.70
TTO	0.63 (0.35)	0.73 (0.37)	.64
Olfactory‐specific QoL, mean (SD)			
QOD‐NS + PS	28.89 (13.62)	20.10 (14.26)	.11
SNOT‐22	29.84 (21.10)	52.40 (22.78)	.01*
SNOT‐22 rhinologic domain	7.53 (4.87)	16.5 (4.65)	<.001**

*Note*: *p < .05; **p < .01 (two‐tailed).

Abbreviations: CRS/R, chronic rhinosinusitis or rhinitis; EQ‐VAS, EuroQol‐Visual Analog Scale; EQ‐5D, EuroQol‐5 dimension; HUV, health utility value; QOD‐NS + PS, questionnaire of olfactory disorders negative statements and positive statements; SNOT‐22, sino‐nasal outcome test; SD, standard deviation; TTO, time trade‐off; VAS, visual analog scale of subjective olfaction.

### 3.3 Subjective olfactory and sinonasal related quality of life assessments

Subjective smell rating via VAS method showed no significant difference in severity of smell loss between COVID‐19 and CRS/R groups in both overall cohort (VAS, COVID‐19: 43.06 ± 32.79 vs. CRS/R: 34.29 ± 25.88, *p* = .35) **(**Table 2 **)** and objectively tested cohort (VAS, COVID‐19: 41.67 ± 28.75 vs. CRS/R: 30.00 ± 25.39, *p* = .38) **(**Table 3 **)**.

**FIGURE 4:**
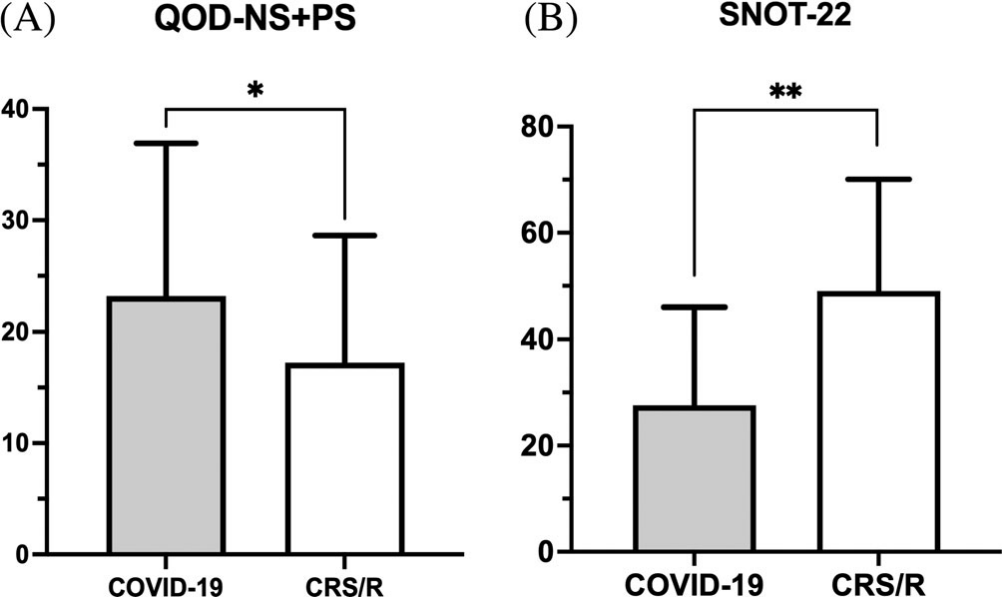
Comparison of mean (a) QOD‐NS + PS and (b) SNOT‐22 among all subjects with self‐reported OD. OD, olfactory dysfunction; QOD‐NS + PS, questionnaire of olfactory disorders – negative and positive statements; SNOT‐22, sino‐nasal outcome test. *p < .05, **p < .01

The mean comparison of QOD‐NS + PS and SNOT‐22 scores between COVID‐19 and CRS/R with OD groups is displayed in Figure 4. Subjects with COVID‐19 OD reported a greater negative impact on olfactory specific QoL compared to those with CRS/R OD (QOD‐NS + PS, COVID‐19: 23.18 ± 13.73 vs. CRS/R: 17.25 ± 11.38, *p* = .04). In contrast, CRS/R OD subjects reported higher average SNOT‐22 scores suggesting worse sinonasal specific QoL compared to COVID‐19 OD subjects (SNOT‐22, CRS/R: 49.03 ± 21.04 vs. COVID‐19: 27.58 ± 18.45, *p* < .001). Since SNOT‐22 is a clinically validated tool to measure sinonasal quality‐of‐life and not specific to COVID‐19 patients, we selected only the rhinologic domain of SNOT‐22 to repeat comparison analysis and the result was consistent with analysis of overall SNOT‐22 **(**Tables 2 and 3 **).**

**FIGURE 5:**
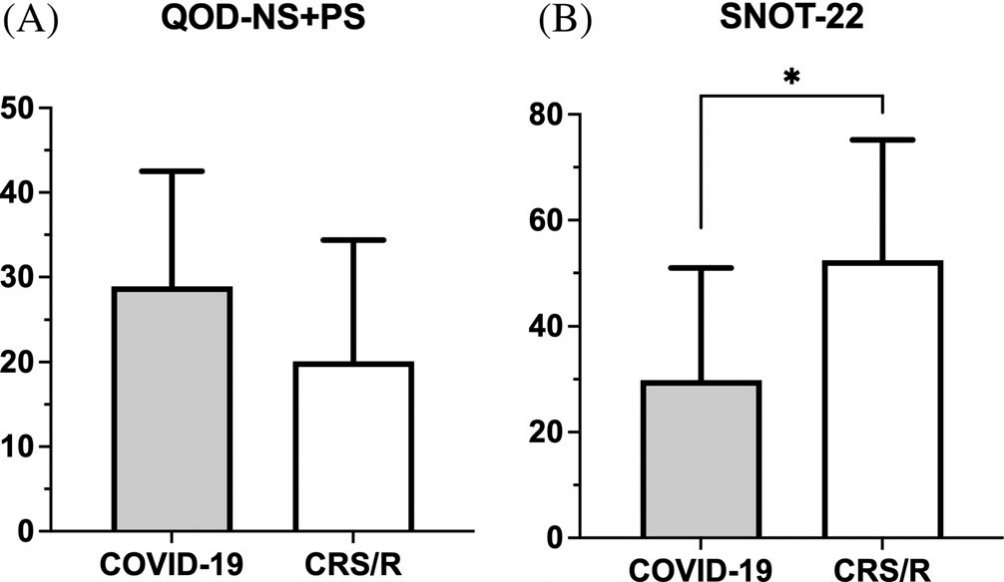
Comparison of mean (a) QOD‐NS + PS and (b) SNOT‐22 among subjects with quantifiably measured OD as confirmed by UPSIT or BSIT. BSIT, brief smell identification test; OD, olfactory dysfunction; QOD‐NS + PS, questionnaire of olfactory disorders – negative and positive statements; SNOT‐22, sino‐nasal outcome test; UPSIT, University of Pennsylvania smell identification test. *p < .05

Those with objectively measured OD reported similar olfactory / sinonasal QoL scores compared to the entire cohort. The mean SNOT‐22 score in the CRS/R group remained higher than the COVID‐19 group (SNOT‐22, CRS/R: 52.40 ± 22.78 vs. COVID‐19: 29.84 ± 21.10, *p* = .01) while the olfactory‐specific mean QOD values were lower than the COVID‐19 group though not statistically significant (QOD‐NS + PS, CRS/R: 20.10 ± 14.26 vs. COVID‐19: 28.89 ± 13.62, *p* = .11) **(**Table 3 **)**. Comparison of mean QOD‐NS + PS and SNOT‐22 among subjects with quantifiably measured OD is demonstrated in Figure 5.

### 3.4 Multivariate linear regression analysis

Multivariate regression analysis was performed to adjust for the potential confounding effects of age, gender, and duration of smell loss on QoL.^21^, ^22^, ^23^, ^24^, ^25^ Results retained similar trends to means comparison analysis (without adjustments) between COVID‐19 and CRS/R with worse EQ‐VAS(*p* = .03), EQ‐5D (*p* = .63), SNOT‐22 (*p* < .001), and SNOT‐22 rhinologic symptoms domain (*p* < .001) scores in the CRS/R cohort and worse TTO (*p* = .52), QOD‐NS + PS (*p* = .02) scores in the COVD‐19 cohort (Table S1).

### 3.5 Spearman's correlations among quality of life instruments

Spearman correlation coefficients were calculated to further characterize the relationship between HUV measures (EQ‐VAS, EQ‐5D, TTO) vs. olfactory and sinonasal‐specific QoL assessments (QOD‐NS + PS and SNOT‐22) between the two groups **(**Table 4). Higher QOD‐NS + PS and SNOT‐22 scores indicate higher disease burden, while lower HUV scores indicate worse QoL. No significant correlation was found between EQ‐VAS and QOD‐NS + PS scores in either the COVID‐19 (*r* = 0.013, *p* = 0.914) or the CRS/R group (*r* = −0.143, *p* = 0.467). Similarly, EQ‐5D was not correlated with QOD‐NS + PS in either group (COVID‐19: *r* = −0.091, *p* = 0.443; CRS/R: *r* = −0.164, *p* = 0.370). TTO was weakly correlated with QOD‐NS + PS in COVID‐19 group (*r* = −0.341, *p* = 0.007), but not in CRS/R group (*r* = −0.166, *p* = 0.429).

**TABLE 4:** Spearman's correlations between overall general health utility and the sinonasal‐specific (SNOT‐22) and olfactory‐specific (QOD‐NS + PS) QoL assessments

	EQ‐VAS		EQ‐5D		TTO	
	COVID‐19	CRS	COVID‐19	CRS/R	COVID‐19	CRS/R
QOD‐NS + PS	0.013 (0.914)	−0.143 (0.467)	−0.091 (0.443)	−0.164 (0.370)	−0.341 (0.007)**	−0.166 (0.429)
SNOT‐22	−0.314 (0.008)**	−0.453 (0.014)*	−0.275 (0.019)*	−0.538 (0.001)**	−0.024 (0.856)	−0.263 (0.195)

*Note*: Table shows Spearman's correlation coefficient *r* (*p*‐value). *p < .05; **p < .01 (two‐tailed).

Abbreviations: CRS/R, chronic rhinosinusitis or rhinitis; EQ‐VAS, EuroQol‐visual analog scale; EQ‐5D, EuroQol‐5 dimension; QOD‐NS + PS, questionnaire of olfactory disorders negative statements and positive statements; QoL, quality of life; SNOT‐22, sino‐nasal outcome test; TTO, time trade‐off.

EQ‐VAS score was weakly but significantly correlated with SNOT‐22 scores among those suffering from COVID‐19 (*r* = −0.314, *p* = 0.008) and moderately correlated with SNOT‐22 scores among those with CRS/R (*r* = −0.453, *p* = 0.014). Similarly, EQ‐5D score was correlated with SNOT‐22 scores in both COVID‐19 (*r* = −0.275, *p* = 0.019) and CRS/R group (*r* = −0.538, *p* = 0.001). There was no correlation between TTO and SNOT‐22 in either COVID‐19 (*r* = −0.024, *p* = 0.856) or CRS/R (*r* = −0.263, *p* = 0.195) group.

## 4 DISCUSSION

This study investigates the impact of COVID‐19 and CRS/R induced OD on health utility values and olfactory related QoL. Our study corroborates the negative impact of OD associated with both COVID‐19 and CRS/R on QoL, as demonstrated by the lower HUV (with EQ‐VAS and ED‐5D) compared to that of reported age‐matched US population norms.

Health utility value between COVID‐19 and CRS/R induced OD were evaluated using multiple assessments (EQ‐VAS, EQ‐5D, TTO). The self‐perceived HUV for this study's COVID‐19 associated OD cohort was consistent with previously reported HUV data from our team's cross‐sectional study in a similar patient population.^6^ In addition, our CRS/R‐associated OD group reported EQ‐VAS and TTO health values comparable to the CRS populations (with unknown olfactory status) described in a prior study by Ference et al.^8^

Comparisons of different HUV methods showed no difference in average HUV scores in indirect measurement of HUV, via EQ‐5D between COVID‐19 and CRS/R group. However, direct measurements showed a significant difference between the two groups via EQ‐VAS method, and a trend toward significance via TTO method. Notably, EQ‐VAS scores were worse in the CRS/R cohort whereas TTO assessment demonstrated worse scores in the COVID‐19 cohort. While both VAS and TTO are direct assessments of HUVs, the EQ‐VAS instrument is a more global assessment of health whereas the TTO instrument is more aligned with olfactory‐specific QoL measurements given that the latter instrument addresses the years one would trade for perfect smell function. This could explain the difference in the impact of OD on QoL between COVID‐19 and CRS/R group. These findings were independent of subjective and objective smell loss severity.

When evaluating olfactory‐specific QoL using QOD measurements, OD related to COVID‐19 had a worse impact on QoL scores compared to CRS/R. This finding suggests that the chronic, gradual onset and fluctuation of CRS/R symptoms may contribute to better adjustment and coping mindset in CRS/R subjects. In addition, current understanding of clinical factors associated with olfactory recovery in COVID‐19 patients with persistent OD is still limited.^26^, ^27^ This lack of understanding of prognosis combined with its acute onset may have contributed to the more severe psychological impact of OD and coping potential assessed by QOD‐NS + PS method in our COVID‐19 group.

On the other hand, the EQ‐VAS assessment is also impacted by the heterogeneity of symptoms associated with CRS/R that result in worse health scores for these patients. Similarly, CRS/R induced OD has a more severe impact on sinonasal specific QoL outcomes compared to COVID‐19 induced OD in both our overall and subgroup analysis among those with objective OD on the SNOT‐22. While the majority of subjects with COVID‐19 induced OD had recovered from the acute phase of the COVID‐19 infection with mainly rhinologic symptoms at the time of enrollment, subjects with CRS/R often presented with other nasal, pain, sleep, and psychiatric related symptoms in addition to smell loss that queried by the SNOT‐22. Thus, worse SNOT‐22 scores likely reflect the presence and severity of multiple non‐olfactory symptoms commonly experienced by subjects with CRS/R induced OD. ^13^

Although there is no clinically validated method to specifically assess QoL in COVID‐19 patients with OD, we believe that overall SNOT‐22 score can be used to aid in the initial quantitative evaluation of the impact of OD on QoL in post‐COVID‐19.^28^, ^29^, ^30^ Our analysis using total SNOT‐22 was consistent with analysis of the rhinologic domain of SNOT‐22. Furthermore, a previously published study has showed the correlation between the overall SNOT‐22 scores with COVID‐19 symptoms such as hyposmia and duration of olfactory dysfunction.^28^ For this reason, we believe that the use of SNOT‐22 is helpful to quantitatively characterize the severity of post COVID‐19 related OD, potentially contributing to the development of an appropriate method to assess QoL related to OD in post COVID‐19 patients as the pandemic remains unsettled worldwide.

Since higher QOD‐NS + PS and SNOT‐22 scores indicate higher disease burden and lower HUV scores indicate worse overall health status, we expected a negative correlation between HUVs and QOD‐NS + PS/ SNOT‐22. Similar to previous studies^8^, ^31^, ^32^ of health utility assessments among all CRS patients, we observed a weakly negative but significant correlation between EQ‐VAS and SNOT‐22 among CRS patients. The correlation was weaker between SNOT‐22 and EQ‐VAS in the COVID‐19 cohort which had a lower sinonasal specific symptomatic burden. Interestingly, the QOD‐NS + PS and the general health EQ‐VAS scores were not correlated in the COVID‐19 OD group despite worse scores in both compared to norms. However, QOD‐NS + PS was correlated with TTO in this group. Thus, careful evaluation of olfactory‐specific QoL outcome measures is important to assess improvement in this patient group as they may represent more granular and sensitive changes in patient clinical outcomes.

Limitations to this study include a small sample size of subjects, particularly in the CRS/R group with 35 individuals. In addition, there is a small sample of subjects with objectively confirmed OD. Sixty‐five percent (30/46) of subjects who underwent objective olfactory testing had scores in the hyposmic range, similar to previous study which reported the difference between self‐perceived and objectively measured olfactory performance.^25^ However, our sub‐analysis of only participants with objectively confirmed OD showed results that were consistent with overall analysis of participants with self‐perceived OD. Moreover, while most subjects in COVID‐19 group took the UPSIT, most subjects in CRS/R group took the shorter smell test – BSIT. Despite different methods of objective evaluations between two groups, both tests have been well studied and validated.^15^, ^18^ We were unable to perform power analysis prior to the study due to the limited available data, the heterogeneity of assessment methods as well as population characteristics among published studies in the literature. Further, the recruitment of subjects from a single academic center may reflect a selection bias as those electing to participate in this study may suffer from more severe OD and/or have comorbidities.

In conclusion, our study established HUVs associated with OD in CRS/R compared to OD in COVID‐19, which could be used as baseline to assess the efficacy of therapeutic interventions in improvements in this domain. In addition, this study showed that CRS/R induced OD has a more severe impact on general health utility and sinonasal specific QoL outcomes, while COVID‐19 induced OD has a greater burden on olfactory‐specific QoL. Comparing QoL assessments associated with OD aids in our understanding about the aspects of QoL that are more heavily impacted by each condition, which helps guide treatment algorithms in these diseases. Further larger scale studies are needed to characterize the impact of OD severity on QoL.

## CONFLICT OF INTEREST

Adam S. DeConde is a consultant for Stryker Endoscopy and receives speaker's fees for GSK.

## Supporting information


**TABLE S1** Comparison analysis of QoL measurements between COVID‐19 and CRS/R group, adjusted for covariates age, gender, duration of smell loss
