# Supplementary material for: Impact of COVID‐19 versus chronic rhinosinusitis/rhinitis associated olfactory dysfunction on health utility and quality of life
Source: Laryngoscope Investig Otolaryngol. 2022 Sep 24;7(5):1299–307. doi: 10.1002/lio2.921 (PMC9538416; doi:10.1002/lio2.921)
Supplement: Supplementary file 1 — TABLE S1 Comparison analysis of QoL measurements between COVID‐19 and CRS/R group, adjusted for covariates age, gender, duration of smell loss [file LIO2-7-1299-s001.docx]

**Title:** Impact of COVID-19 versus chronic rhinosinusitis/rhinitis associated olfactory dysfunction on health utility and quality of life

**Authors:** Thanh Luong, BS; Sophie S Jang, MD; Mena Said, MD; Adam S. DeConde, MD; Carol H. Yan MD

**_____________________________________________________________________________________**

**Supplement Table 1.** Comparison analysis of QoL measurements between COVID-19 and CRS/R group, adjusted for covariates age, gender, duration of smell loss

|  | **B** | **Std. Error** | **t** | ***p*-value** | **95% CI** | |  |
| --- | --- | --- | --- | --- | --- | --- | --- |
|  |  |  |  |  | **Lower Bound** | **Upper Bound** |  |
| **EQ-VAS** | | | | | | |  |
| Intercept | 0.642 | 0.078 | 8.201 | <0.001 | 0.487 | 0.798 |  |
| CRS/R | ref |  |  |  |  |  |  |
| COVID-19 | 0.124 | 0.057 | 2.177 | 0.032* | 0.011 | 0.238 |  |
| **EQ-5D** | | | | | | |  |
| Intercept | 0.840 | 0.043 | 19.542 | <0.001 | 0.755 | 0.925 |  |
| CRS/R | ref |  |  |  |  |  |  |
| COVID-19 | 0.015 | 0.032 | 0.480 | .631 | -0.048 | 0.079 |  |
| **TTO** | | | | | | |  |
| Intercept | 0.685 | 0.149 | 4.610 | <0.001 | 0.389 | 0.980 |  |
| CRS/R | ref |  |  |  |  |  |  |
| COVID-19 | -0.068 | 0.106 | -0.648 | 0.519 | -0.279 | 0.142 |  |
| **QOD-NS+PS** |  |  |  |  |  |  |  |
| Intercept | 20.037 | 4.636 | 4.322 | <0.001 | 10.837 | 29.237 |  |
| CRS/R | ref |  |  |  |  |  |  |
| COVID-19 | 8.049 | 3.450 | 2.333 | 0.022* | 1.203 | 14.896 |  |
| **SNOT-22** | | | | | | |  |
| Intercept | 60.903 | 7.250 | 8.401 | <0.001 | 46.512 | 75.293 |  |
| CRS/R | ref |  |  |  |  |  |  |
| COVID-19 | -23.934 | 5.454 | -4.388 | <0.001** | -34.759 | -13.108 |  |
| **SNOT-22 Rhinologic Domain** |  |  |  |  |  |  |  |
| Intercept | 18.890 | 1.828 | 10.331 | <0.001 | 15.261 | 22.519 |  |
| CRS/R | ref |  |  |  |  |  |  |
| COVID-19 | -9.270 | 1.375 | -6.739 | <0.001** | -12.000 | -6.540 |  |
|  |  |  |  |  |  |  |  |

*p<0.05, **p<0.01 (2-tailed).

EQ-VAS = EuroQol-Visual Analog Scale; EQ-5D = EuroQol-5 Dimension; TTO = Time Trade-Off

QOD-NS+PS = Questionnaire of Olfactory Disorders Negative Statements and Positive Statements; SNOT-22 = Sino-Nasal Outcome Test; CRS/R = chronic rhinosinusitis or rhinitis
